# Synergistic potential of stem cells and microfluidics in regenerative medicine

**DOI:** 10.1007/s11010-024-05108-8

**Published:** 2024-09-16

**Authors:** Resmi Rajalekshmi, Devendra K. Agrawal

**Affiliations:** Department of Translational Research, Western University of Health Sciences, 309 E. Second Street, Pomona, CA 91766 USA

**Keywords:** Regenerative medicine, Microfluidics, Stem cells, Tissue repair, Biomedical devices

## Abstract

Regenerative medicine has immense potential to revolutionize healthcare by using regenerative capabilities of stem cells. Microfluidics, a cutting-edge technology, offers precise control over cellular microenvironments. The integration of these two fields provides a deep understanding of stem cell behavior and enables the development of advanced therapeutic strategies. This critical review explores the use of microfluidic systems to culture and differentiate stem cells with precision. We examined the use of microfluidic platforms for controlled nutrient supply, mechanical stimuli, and real-time monitoring, providing an unprecedented level of detail in studying cellular responses. The convergence of stem cells and microfluidics holds immense promise for tissue repair, regeneration, and personalized medicine. It offers a unique opportunity to revolutionize the approach to regenerative medicine, facilitating the development of advanced therapeutic strategies and enhancing healthcare outcomes.

## Introduction

Regenerative medicine holds the promise of transforming healthcare by harnessing the inherent regenerative capabilities of the human body. At the forefront of this revolutionary field, stem cells emerge as key players with their ability to differentiate into various cell lineages, offering a regenerative potential that extends across diverse tissues and organs [1]. Concurrently, microfluidics, a cutting-edge technology, has gained prominence for its precision in manipulating fluids at the microscale [2]. The integration of stem cells and microfluidics presents a paradigm shift in regenerative medicine, offering unprecedented control over the cellular microenvironment and opening avenues for innovative therapeutic strategies.

Stem cells, characterized by their self-renewal and differentiation capabilities, constitute a foundational element in regenerative medicine. Embryonic stem cells, adult stem cells, and induced pluripotent stem cells each bring unique attributes to the regenerative landscape [3]. The ability of stem cells to differentiate into specialized cell types holds immense potential for tissue repair, regeneration, and personalized medicine. Yet, harnessing this potential requires a thorough understanding of the intricate interplay between stem cell behavior and the microenvironment in which they reside.

Microfluidics, with its roots in miniaturized fluid-handling systems, has evolved into a powerful tool for precisely controlling cellular microenvironments. The ability to manipulate fluids on the scale of micrometers allows for the creation of intricate systems that mimic the physiological conditions of tissues and organs. Microfluidic platforms offer controlled nutrient supply, precise mechanical stimuli, and real-time monitoring, providing an unprecedented level of detail in studying cellular responses [4]. This technology stands poised to revolutionize our approach to regenerative medicine by facilitating a deeper understanding of stem cell behavior and enabling the development of advanced therapeutic strategies.

This comprehensive review explores the convergence of stem cells and microfluidics, delving into the synergistic potential that emerges when these two fields intersect. We will examine the utilization of microfluidic systems to culture and differentiate stem cells with unparalleled precision. From biomimetic microenvironments to organ-on-a-chip models, this convergence promises to unlock novel insights into stem cell biology and tissue regeneration.

## Basics of stem cells in regenerative medicine

Stem cells represent a unique category of cells with the remarkable ability for sustained self-renewal through replication and the potential to differentiate into specific tissue types [5]. Initially identified by McCulloch and colleagues in 1963 [6], stem cells have become increasingly prominent due to their pivotal roles in various domains, including tissue engineering [7], organ regeneration [8], cell-based therapies [9], disease modeling [10] and drug development [11]. Their capacity for continuous self-renewal and differentiation has made stem cells invaluable in addressing challenges such as healing damaged tissues and replacing nonfunctional organs. The discovery of stem cells has opened avenues for transformative advancements in medical science and holds great promise for enhancing healthcare outcomes.

### Types of stem cells

Stem cells, classified as totipotent, pluripotent, or multipotent, can be extracted from the human body, offering diverse differentiability into various cell types. Totipotent stem cells originate from the zygote, while pluripotent stem cells, derived from primitive germ line stem cells, can differentiate into cardiac cells, neural cells, and blood cells. Multipotent stem cells, found in adults, give rise to a limited number of specific cell types.

#### Embryonic stem cells (ESCs)

Derived from the blastocyst’s inner cell mass, embryonic stem cells (ESCs) exhibit pluripotency, enabling differentiation into cells representing all three germ layers [12]. Serving as invaluable tools, ESCs unravel intricate developmental mechanisms, offering insights into specialized cell development and organ structure establishment. Their intrinsic indefinite self-renewal and plasticity provide an in vitro platform for generating diverse cell types, fostering novel avenues in regenerative medicine.

The therapeutic potential of human ESCs (hESCs) lies in their capability to replace damaged tissue in individuals with degenerative diseases [13]. However, ongoing investigations focus on understanding signaling mechanisms that orchestrate ESC lineage restriction toward distinct cellular phenotypes [14]. Propelling hESC-based therapies into clinical applications necessitates developing appropriate culture conditions, ensuring the generation of genetically stable, homogeneous cell populations to mitigate potential adverse effects post-transplantation.

A study investigated the effectiveness of hESCs versus bone marrow-derived mesenchymal stem cells (BM-MSCs) in mice with chemotherapy-induced premature ovarian failure (POF). After treatment with cyclophosphamide and busulfan, mice received transplants of either cell type. Both hESCs and BM-MSCs significantly improved hormone secretion, survival rates, reproductive function, and reduced follicular apoptosis, enabling new offspring production. The study concluded that hESCs were as effective as BM-MSCs in repairing ovarian function through enhanced follicular development and paracrine effects [15].

While ESCs are pivotal tools in biology with the capacity to theoretically differentiate into any cell type, challenges such as ethical concerns about human embryo destruction for hESC generation persist [16]. Additionally, a significant barrier involves the need for Human Leukocyte Antigen (HLA)-compatible hESC lines, hindering their medical use [17]. Overcoming these challenges is vital for unlocking the full potential of ESCs in advancing regenerative medicine. Pluripotent stem cells derived from ESCs play crucial roles in drug development, screening, and cell toxicology [18]. Notably, nuclear transfer technology facilitates the generation of ESC lines harboring genetic disease markers, aiding the study of underlying disease causes.

Despite the vast potential, challenges persist, particularly in ESC-induced cell therapy. Concerns such as transplant rejection and ethical considerations demand careful integration of technological advancements and ethical frameworks. ESCs, prone to rapid differentiation without optimal conditions or genetic manipulation, require thoughtful consideration in addressing challenges related to transplant rejection and navigating ethical concerns. Achieving this delicate balance is imperative to unlock the full therapeutic potential of ESCs in regenerative medicine.

#### Adult stem cells (ASCs)

**Fig. 1:**
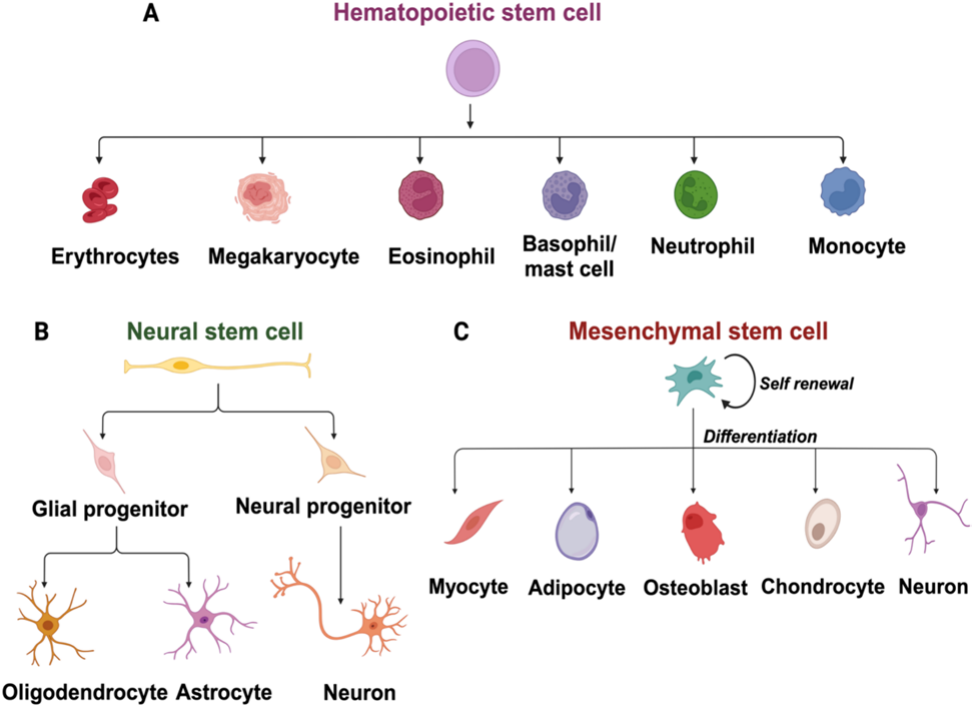
Different types of adult stem cells and their differentiation potential

Found within somatic adult tissues, tissue-specific stem cells or adult stem cells, play a pivotal role in maintaining tissue homeostasis and facilitating repair processes [19]. Distinguishing themselves from typical somatic cells, adult stem cells do not actively contribute to the regular functioning of tissues but instead, function as a reserve of cells capable of generating various highly specialized cell types. As individuals age or experience disease, the quantity and/or functionality of adult stem cells decrease, impeding the replacement of aged or dysfunctional cells and thereby contributing to the diminished overall performance of tissues [20]. Adult organisms harbor diverse types of stem cells, including mesenchymal stem cells (MSCs), hematopoietic stem cells (HSCs), and neural stem cells (NSCs), all considered multipotent due to their capacity to generate mature cell types within specific lineages (Fig. 1A–C) [21]. Unlike embryonic stem cells, adult stem cells cannot reconstitute the entire organism [22]. The versatility of adult stem cells is evident in various tissues, such as the brain, bone marrow, peripheral blood, blood vessels, skeletal muscle, skin, teeth, heart, liver, ovaries, and epithelium [23]. Their ethical derivation from peripheral tissues has positioned them as key players in regenerative medicine.

MSCs from adults exhibit differentiation potential into osteoblasts [24], adipocytes [25], chondrocytes [26], astrocytes [27], tenocytes [28] and myoblasts [29]. Additionally, MSCs from different sources, including adult marrows [30], cord blood [31], placentas [32], and amniotic fluids [33], demonstrate the ability to differentiate into cell types from ectoderm, mesoderm, and endoderm. A recent study found that treatment BM-MSC significantly improved colitis symptoms and inflammation in a mouse model. It also restored enteric neuronal density and provided neuroprotection, suggesting its potential as a therapy for chronic colitis [34].

HSCs, crucial for replenishing all blood cell types, are conveniently sourced from cord blood, providing a readily available and noninvasive collection method with lower cytomegalovirus infection risk. NSCs, similar to HSCs, possess the ability to differentiate into various cell types, particularly those in the immune system. They self-renew and generate neurons, astrocytes, and oligodendrocytes, holding therapeutic promise for neurodevelopmental and neurodegenerative diseases like Parkinson’s and Alzheimer’s [35]. This collective knowledge enhances our understanding of various physiological processes and holds potential for therapeutic applications in regenerative medicine.

#### Induced pluripotent stem cells (iPSCs)

Induced pluripotent stem cells (iPSCs) demonstrate an extraordinary capacity for continuous self-renewal in culture, coupled with the ability to differentiate into diverse specialized cell types [36]. Unlike naturally occurring cells, iPSCs are generated or "induced" through ectopic co-expression of defined pluripotency factors in culture,

originating from somatic cells [37]. This reprogramming process offers a valuable resource for regenerative medicine, enabling the replacement of diseased or damaged tissues. iPSCs, accessible from any healthy individual or patient, have emerged as a versatile tool for studying cell fate decisions, modeling human diseases, and advancing drug discovery and therapeutic strategies [38].

Despite their commonalities with embryonic stem cells, iPSCs possess distinct properties that researchers are actively exploring to understand their biological implications. Initial production of iPSCs involved the use of viruses to introduce extra copies of genes into tissue-specific cells [39]. Ongoing research explores alternative approaches for creating iPSCs, emphasizing their potential as a cellular source for medical treatments. This progress marks a breakthrough in stem cell research, providing a method to obtain pluripotent stem cells without resorting to the controversial use of embryos.

The advent of iPSCs opens avenues for “de-differentiating” cells whose developmental fates were conventionally assumed to be fixed. Tissues derived from iPSCs closely match the genetic makeup of the cell donor, enhancing their utility in disease modeling and drug screening [40]. The potential of iPSCs extends to reprogramming cells for the repair of damaged tissues within the human body [41]. A recent study used human induced pluripotent stem cell-derived β islet-like cells to treat type 1 diabetes. They employed "human elastin-like recombinamers" (ELRs) to improve cost and differentiation efficiency. ELRs provided immunoprotection without compromising cell viability or function. Initial in vivo results suggest that ELRs protect cell grafts, and further research is ongoing to evaluate their potential in correcting hyperglycemia [42].

This scientific advancement not only addresses ethical concerns associated with embryonic stem cells but also positions iPSCs as a promising tool for transformative developments in regenerative medicine and biomedical research.

## Current challenges and opportunities in stem cell therapies

The field of stem cell therapies is marked by significant progress and promising developments, yet it also faces various challenges that need to be addressed. Understanding these challenges and opportunities is crucial for advancing the field and realizing the full potential of stem cell-based interventions.

### Immunological rejection

Immunological rejection remains a substantial challenge in the field of stem cell therapies, particularly when utilizing allogeneic stem cells [43]. The recipient’s immune system may recognize transplanted cells as foreign, leading to rejection. Researchers have explored strategies like immunosuppression to modulate the immune response. However, inhibiting immune responses, through anti-inflammatory or immunosuppressive agents, is often ineffective or associated with significant side effects due to the crucial role of the immune response in the regeneration process [44].

Autologous cells, derived from the patient’s own body, represent an alternative approach to mitigate the risk of rejection [45]. Creating stem cell banks tailored to diverse populations emerges as a solution to enhance the availability of compatible cells for transplantation, effectively addressing concerns related to compatibility and minimizing the likelihood of immune rejection. The proposed strategy involves the establishment of stem cell banks that consist of HLA-typed human embryonic stem cells (hESCs) and induced pluripotent stem cells (iPSCs) [46]. This approach aims to overcome immunological barriers by offering histocompatible tissue that matches the HLA profile of the target population.

### Tumorigenicity

Concerns regarding the tumorigenic potential of pluripotent stem cells, including ESCs and iPSCs, necessitate comprehensive safety measures. The inherent qualities of self-renewal and pluripotency, which contribute to the therapeutic promise of these cells, also underlie their significant tumorigenic potential [47]. This tumorigenicity can be categorized into two distinct types: the malignant transformation of differentiated iPSCs and the formation of benign teratomas from remaining undifferentiated iPSCs [48]. These processes can give rise to tumors composed of either specific cell types or all three germ layers, respectively.

Rigorous screening and monitoring protocols have been implemented to minimize the risk of tumor formation associated with stem cell therapies. Advances in genetic engineering and molecular biology techniques, such as CRISPR-Cas9, contribute to enhancing the safety profile of stem cells and reducing the potential for tumorigenesis [49].

### Ethical considerations

The ethical considerations surrounding stem cell sources are intricate and contentious. The identification of multipotent stem cells, including human embryonic stem cells, has introduced novel ethical and political dilemmas in stem cell research. Concerns have been raised about the ethical implications of obtaining multipotent stem cells through the destruction of embryos [50]. Although induced pluripotent stem (iPS) cells are functionally similar to embryonic stem cells and seemingly avoid the ethical issues associated with their use, ethical dilemmas persist. The genetic manipulations involved in deriving iPS cells raise concerns about potential risks, including toxicity, tumorigenicity, and immunological reactions [51]. Furthermore, the safety concerns associated with iPSC transplantation necessitate rigorous optimization of iPSC differentiation protocols to ensure the purity of iPSC-derived cell populations [52]. Moreover, issues like regulatory arbitrage, regulatory brokerage, stem cell hype, and changes in moral status require careful consideration [53]. This underscores the complex ethical landscape surrounding stem cell research and the need for careful consideration of the ethical implications associated with different stem cell sources. Monitoring and long-term follow-up studies are imperative for MSC-based therapies, given the potential for promoting tumor growth and metastasis, highlighting the need for continuous scrutiny in MSC-treated animal models to identify possible detrimental effects.

### Regulatory frameworks

As the number of clinical trials employing stem cells for therapeutic purposes increases, the necessity of establishing regulatory guidelines and standards to ensure patient safety becomes increasingly crucial. However, the relatively novel nature of stem cell therapy renders it susceptible to scientific, ethical, and legal controversies that are yet to be fully regulated. Unlike traditional pharmaceutical products, enforcing strict standards for the purity of cell-based products may be unrealistic and even undesirable in cases where a mixture of cells proves beneficial. Leading countries in the field, including the United States and Japan, have taken steps to address these challenges through the formulation of regulatory guidelines. The Food and Drug Administration (FDA) in the United States has released guidelines stipulating that stem cell treatments that have undergone minimal manipulation and are intended for homogeneous use may proceed without premarket approval, subject only to regulations against disease transmission [54]. In Japan, a significant regulatory reform in 2014 introduced conditional approval of cell-based treatments following early phase clinical trials, with treatments classified based on risk and eligible for “fast-track approvals” [55]. Regulatory authorities worldwide are increasingly emphasizing the application of standardization and safety protocols for cellular products, including the use of Xeno-free culture media, recombinant growth factors, and adherence to Good Manufacturing Practice (GMP) standards [56]. Despite limited global regulations, influential bodies such as the FDA, the International Society of Stem Cell Research (ISSCR) [57], and the National Academies of Sciences, Engineering, and Medicine have provided guidance [58]. Collaborative efforts between regulatory agencies, researchers, and industry stakeholders are essential to streamline and harmonize regulations while fostering an environment conducive to innovation.

### High costs and accessibility

The utilization of stem cell therapies in medical treatment has the potential to bring about a transformative impact. These therapies offer regenerative solutions for a wide array of diseases and injuries. However, their widespread adoption is impeded by several significant factors, chiefly among them being the high costs and limited accessibility. A multitude of challenges contribute to these hurdles, commencing with the intricacies of the production processes involved. These processes are characterized by their labor-intensive nature, necessitating skilled personnel and substantial time investments [59]. Moreover, specialized equipment and cutting-edge facilities, which adhere to stringent standards for sterility and quality control, are mandatory [60, 61]. Consequently, the associated costs soar considerably.

Ensuring the safety and efficacy of stem cell products entails rigorous quality control measures, including testing for purity, potency, and stability [62]. These comprehensive testing processes significantly escalate expenses. Regulatory compliance further adds to the financial burden, as stem cell therapies must undergo extensive preclinical and clinical testing to adhere to stringent oversight requirements for patient safety and therapeutic efficacy. The variability in regulations across different regions may necessitate additional resources for compliance, compounding the associated costs.

Personalized therapies, which frequently involve the use of autologous cells to minimize immune rejection risks, significantly contribute to high costs [63]. This is primarily due to the complexity and individualized nature of production. Small-batch production lacks economies of scale, which further increases costs. The market dynamics, limited patient populations, and high investment costs contribute to maintaining high prices for medications [64].

## The need for advanced platforms like microfluidics

Stem cell therapies hold immense promise for regenerative medicine, but their clinical translation faces substantial challenges that necessitate advanced platforms such as microfluidics. One major obstacle lies in the complexity of guiding stem cell fate decisions, as traditional culture methods often lack the precise control required for reproducible differentiation [65]. Microfluidic systems offer a unique solution by enabling spatiotemporal control over the cellular microenvironment, allowing for the emulation of physiological conditions [66]. Additionally, scalability and automation in microfluidic platforms streamline the production process, addressing challenges associated with large-scale manufacturing of consistent and high-quality stem cell-based products [67]. Furthermore, microscale technologies facilitate the study of stem cell behavior at unprecedented resolutions, contributing to a deeper understanding of cellular responses to microenvironmental cues [68–70]. Overcoming these challenges through the integration of microfluidics into stem cell research and therapy development is crucial for harnessing the full potential of regenerative medicine**.**

## Fundamentals of microfluidics technology

Microfluidics is an interdisciplinary field that deals with the precise manipulation of small quantities of liquids in microchannels with rectangular cross-sections [71]. This emerging technology integrates biology, engineering, and computation to create favorable environments for cells and tissues [72]. Researchers use microfluidic devices and methods to precisely control the microenvironment of cells, enabling culture, maintenance, and analysis under nearly optimum conditions. The technology works at microliter to picoliter levels and exhibits unique effects in micro-domains, with laminar flow and diffusion-dominated mixing being prominent features [73]. The integration of proper mixing configurations within microfluidics facilitates rapid mixing due to the small distances in the channels.

**Fig. 2:**
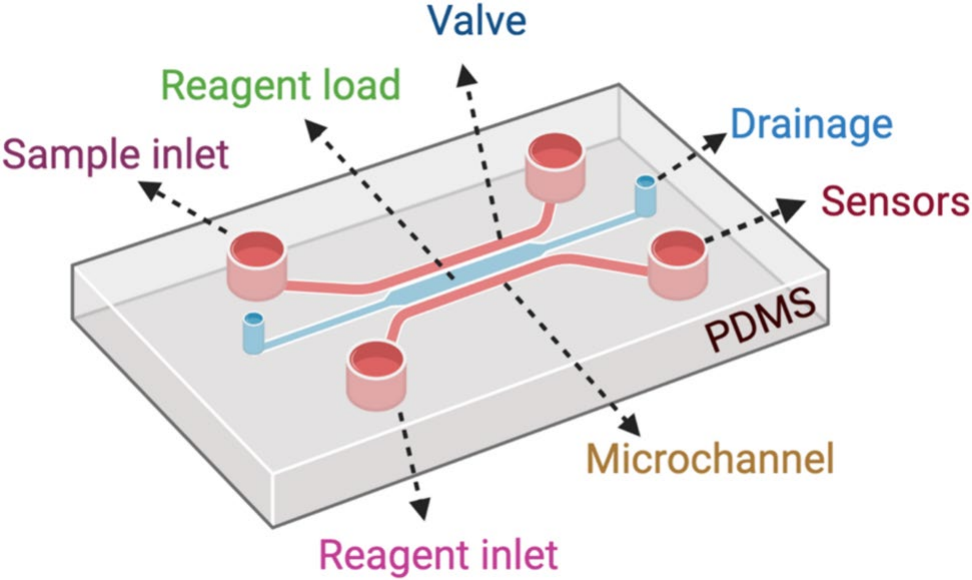
Schematic representation of the microfluidic chip

The applications of microfluidics are diverse, ranging from microarrays to cellular biophysics, and are known as “lab-on-a-chip” technology [74]. These systems usually have a size range of 10–100 µm, allowing for compact designs that increase surface area, resulting in high mass transfer and analytical throughput. The technology reduces sample and reagent requirements, achieving multiplexing and high-throughput screening. Microfluidic devices are designed as miniaturized chips that precisely control the physicochemical reactions of contained fluids. The common materials used for fabrication include polydimethylsiloxane (PDMS), silicon, glass, polycarbonate, and polymethylmethacrylate (PMMA) [75]. The basic chip design includes reagent and sample inlets, valves, grooves or microchannels, a drainage system, and a sensor part. The drainage system disposes of waste material, and the sensor assesses outcomes (Fig. 2) [74].

The prominence of microfluidic chip technology is increasing in biomedical applications, featuring grooves or microchannels engraved on materials like glass, silicon, or polymers such as PDMS and PMMA. The interconnected microchannels within the chip provide desired results, acting as an interface between the macro- and miniature worlds. The chip, coupled with a pump, facilitates the determination of microfluid behavioral changes, enabling various operations simultaneously [76]. The versatility and miniaturization capabilities of microfluidic devices make them invaluable tools in advancing various areas of biomedical research, diagnostics, and therapeutic development [77–79].

## Advantages of microfluidics for stem cell studies

Stem cell research has advanced significantly, and the integration of innovative platforms like microfluidics has become crucial for enhancing precision and efficiency. Microfluidics, involving controlled fluid manipulation at the microscale, provides distinct advantages in the study of stem cells. It enables precise mimicry of in vivo conditions, allowing researchers to study gene interactions governing cellular events [74]. While conventional techniques require large cell numbers, microfluidics is ideal for single-cell analysis, overcoming limitations in studying rare stem cells [80].

Microfluidic devices, manipulating nanoliters of fluids, offer cost-effective and efficient single-cell analysis [81]. Moreover, these devices minimize material loss, making them suitable for studying rare cell populations like stem cells. In the context of tissue engineering and stem cell differentiation, microfluidics provides controlled frameworks for directing cellular fate [82]. The ability to create three-dimensional microenvironments enhances the reproducibility of in vitro models for regenerative medicine applications.

Another advantage of stem cell microfluidics is the high-throughput screening capability. Microfluidic devices can be designed to test multiple compounds or conditions simultaneously, making it possible to identify the most effective treatments for a particular disease or condition quickly [83]. Moreover, their efficient use of reagents and samples not only helps lower research and development costs, making stem cell therapies more affordable and accessible to a broader population, but also facilitates faster drug discovery and development, ultimately reducing the time and cost of bringing new therapies to market.

One of the key benefits of this technology is the ability to create personalized treatment plans that are tailored to the patient’s unique physiology [84]. By utilizing a patient’s own stem cells, researchers can develop treatments that are more effective and less likely to cause adverse reactions.

As the technology evolves, the possibility of constructing an "organ-on-a-chip" emerges, providing a more biomimetic environment for studying complex interactions [85]. In the realm of cell-based assays, microfluidic technologies offer precise control over parameters in the microscale. This control enhances the biological relevance of cell culture models, modeling physiological conditions with high throughput. Microfluidic platforms play a pivotal role in advancing stem cell research, offering unprecedented control, efficiency, and versatility. They bridge the gap between in vitro cell culture environments and the complex features of the in vivo stem cell niche. These advancements contribute to our understanding of stem cells and pave the way for clinical applications in regenerative medicine.

## Microfluidic platforms for stem cell culture

**Fig. 3:**
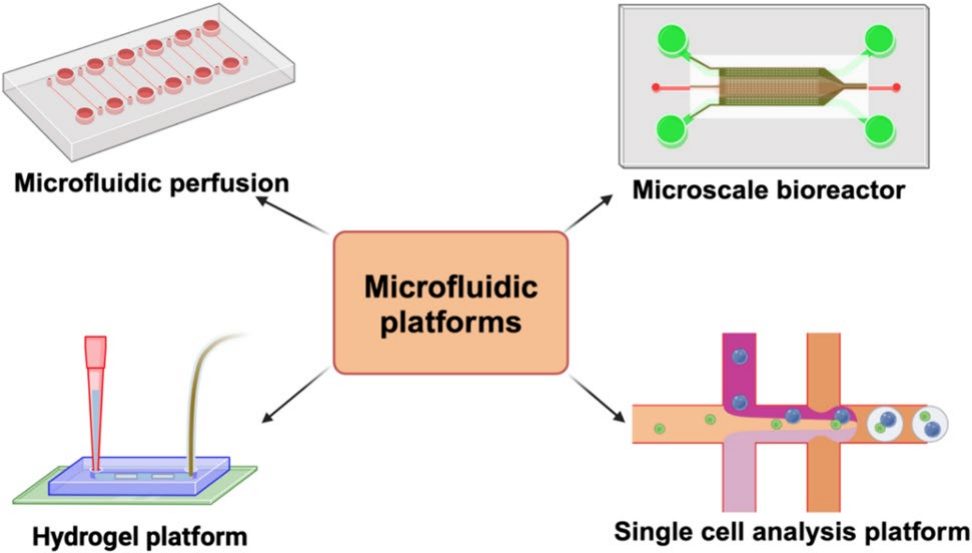
Microfluidic platforms

Microfluidic platforms for stem cell culture provide controlled environments to study and manipulate stem cells at the microscale. These platforms offer advantages such as precise control over cell microenvironments, reduced reagent consumption, and the ability to mimic in vivo conditions. Choosing the right microfluidic platform for stem cell culture depends on several factors, including the research question being addressed, the type of stem cells being cultured, and the desired experimental outcomes. However, microfluidic platforms are becoming increasingly popular in the field of stem cell research due to their ability to provide greater control over the stem cell microenvironment and enable more precise studies of stem cell biology The different types of microfluidic platforms are described below (Fig. 3).

### Microfluidic perfusion

The regulation of stem cell fate is a crucial aspect of controlling the microenvironment, which is influenced by a variety of factors, including soluble factors. These factors’ concentrations are influenced by the culture medium and autocrine and paracrine mechanisms. Microfluidic perfusion techniques provide an efficient way to control the spatial and temporal profiles of these factors.

A new system called HT-μUPS was developed, allowing for high-throughput microfluidic perfusion and compatibility with acute or chronic all-optical electrophysiology studies. The system consisted of a soft multichannel microfluidic plate cover that could be attached to a 96-well plate and was biocompatible, sterilizable, and reusable. The low shear rate near the bottom of wells did not disrupt the cell layer and did not negatively influence their electrophysiology. Long-term culture with HT-μUPS improved cell viability and optogenetically tracked calcium responses in genetically engineered excitable Human Embryonic Kidney (HEK) cell lines. The system demonstrated the potential to enable cell-based assays for personalized medicine using Human induced pluripotent stem-cell-derived cardiomyocytes (iPSC-CMs) [86].

In another study, the impact of microfluidic perfusion, specifically microfluidic polydimethylsiloxane (PDMS) by soft lithography, on the growth and progenitor motor neuron (pMN) differentiation of mouse embryonic stem cell aggregates (mESCs) was discussed. It was found that the media exchange strategy played a crucial role in modulating cell growth and differentiation of mESCs to pMNs. Continuous media perfusion led to less cell growth and minimal generation of pMNs, likely due to modulation of diffusible autocrine/paracrine signaling rather than nutrient limitations. Differentiation under discontinuous media perfusion promoted pMN differentiation, although not to the level of plate controls. Moreover, the frequency of discontinuous media perfusion and concentrations of small molecules added to the media were identified as critical parameters influencing the extent of pMN differentiation. These findings provided insights into how microfluidic media exchange conditions influenced the growth and differentiation of 3D mESC aggregates. [87].

### Microscale bioreactors

Micro-bioreactors (MBRs) are small-scale bioreactor systems designed for growing mammalian cells and/or tissues in vitro. They provide controllable 3D cell culture conditions that are difficult to achieve using larger bioreactors and are useful in cell-based biomedical research. MBRs allow the use of small amounts of chemical entities and low cell numbers, particularly when primary cell and/or tissue availability is limited. MBRs are frequently amenable to microscopic imaging and other sophisticated analysis techniques, which facilitate cell culture evaluation during bioreactor operation [88]. MBRs have enabled in vitro studies that employ 3D cell culture techniques individually or in combination, identifying relevant parameters for the modulation of cell behavior.

A new approach for human pluripotent stem cell (hPSC) culture was developed, involving the use of lumenized three-dimensional colonies inside microfluidics-assisted core–shell microcapsules. This approach significantly improved viability and expansion rates while maintaining pluripotency. By further tuning capsule size and culture conditions, this method could be scaled up to industrial-scale stirred tank bioreactors and achieved an unprecedented hPSC amplification rate of 277-fold in 6.5 days [89].

In another recent study, the use of fused deposition modeling (FDM) technology was described to produce 3D-printed perfusion bioreactors suitable for the culture of human mesenchymal stromal cells under perfusion for up to 2 weeks. The bioreactors were biologically validated, and microenvironments of various sizes/volumes could be engineered by modulating the 3D-printed bioreactor design. The resulting human microenvironments were further exploited for the maintenance of human hematopoietic stem cells. The study concluded that perfusion bioreactors fit for cell culture could be generated using 3D printing technology and exploited for the 3D modeling of complex stem cell systems [90].

### Hydrogel-based platforms

Hydrogel-based microfluidic platforms have emerged as a promising technology for stem cell culture due to their ability to provide a three-dimensional (3D) environment that closely mimics the extracellular matrix. This innovative approach offers researchers the opportunity to create a more physiologically relevant milieu for stem cell culture, leading to more accurate and reliable experimental results. These platforms are beneficial in that they enable the manipulation of cellular microenvironments, facilitating the study of cell behavior in response to mechanical, chemical, and biological stimuli [91].

A new electro-assisted bioprinting method was developed, capable of printing low-concentration pure GelMA microdroplets with low cost, minimal cell damage, and high efficiency. The application of lower external forces to separate the droplets ensured negligible cell damage during printing. The printed microspheres with 5% w/v GelMA provided a suitable microenvironment for laden bone marrow stem cells. These printed microdroplets were utilized in building micro spheroidal organoids, drug-controlled release, and 3D bioprinting as biobricks. This method demonstrated great potential for use in cell therapy, drug delivery, and organoid building [92].

In another recent study, the challenges in high-throughput cell screening (HTCS) were discussed, and a microfluidic spotting-screening platform (MSSP) was introduced as a promising tool for hydrogel-based HTCS. The MSSP was capable of printing microdroplet spots in minutes and controlling the evaporation rate of nanoliter droplets, providing a stable fabrication platform for hydrogel-microarray-based materials. The platform successfully controlled the adhesion, adipogenic, and osteogenic differentiation behavior of mesenchymal stem cells, and the cost and cycle of the entire experimental process were significantly reduced compared to reported HTCS methods [93].

### Single-cell analysis platforms

The intrinsic heterogeneity of individual cells within a stem cell population plays an essential role in the development and progress of diseases. Therefore, accurate disease diagnosis and treatment require an understanding of the differences between individual cells. However, traditional gene profiling methods often fail to differentiate between individual cells. To address this issue, single-cell sequencing has emerged as a promising technique that can provide data to characterize the intrinsic heterogeneity of individual cells [94]. By using a microfluidic application of single-cell sequencing, complex and rare cell populations can also be identified, which can help gain insights into the underlying mechanisms of various diseases.

The literature highlights a comparative study of six prominent single-cell RNA sequencing (scRNA-seq) methods, which are CEL-seq2, Drop-seq, MARS-seq, SCRB-seq, Smart-seq, and Smart-seq2. The study is based on the data generated from 583 mouse embryonic stem cells. The evaluation of these methods indicates that while Smart-seq2 detects the highest number of genes per cell and across cells, CEL-seq2, Drop-seq, MARS-seq, and SCRB-seq enable quantification of mRNA levels with less amplification noise due to the incorporation of unique molecular identifiers (UMIs). The simulations demonstrate that Drop-seq is more cost-efficient when analyzing large numbers of cells, whereas MARS-seq, SCRB-seq, and Smart-seq2 are more efficient when analyzing fewer cells [95].

Another study describes a microfluidic device that uses dielectrophoresis (DEP) forces to sort stem cells and their differentiation progenies. The device contains a large array of interdigitated electrodes that generate an electric field to manipulate the cell trajectories. The device was tested with human mesenchymal stem cells (hMSC) and their differentiation progenies (osteoblasts) at different flow rates, and a clear separation of the two populations was achieved. The experimental measurements were characterized and evaluated quantitatively, and consistent performance was demonstrated. The microfluidic DEP sorting device was found to be feasible for continuous, label-free sorting of stem cells and their differentiation progenies [96].

In summary, microfluidic platforms have significantly advanced stem cell research by providing precise control over the microenvironment and enabling the study of stem cells at the microscale. These platforms integrate with various methodologies to enhance cell viability and differentiation. Hydrogel-based microfluidic platforms enable the creation of relevant environments, while the integration of single-cell analysis techniques with microfluidics enables high-throughput screening and detailed exploration of cellular heterogeneity.

## Stem cell differentiation in microfluidic environments

**Fig. 4:**
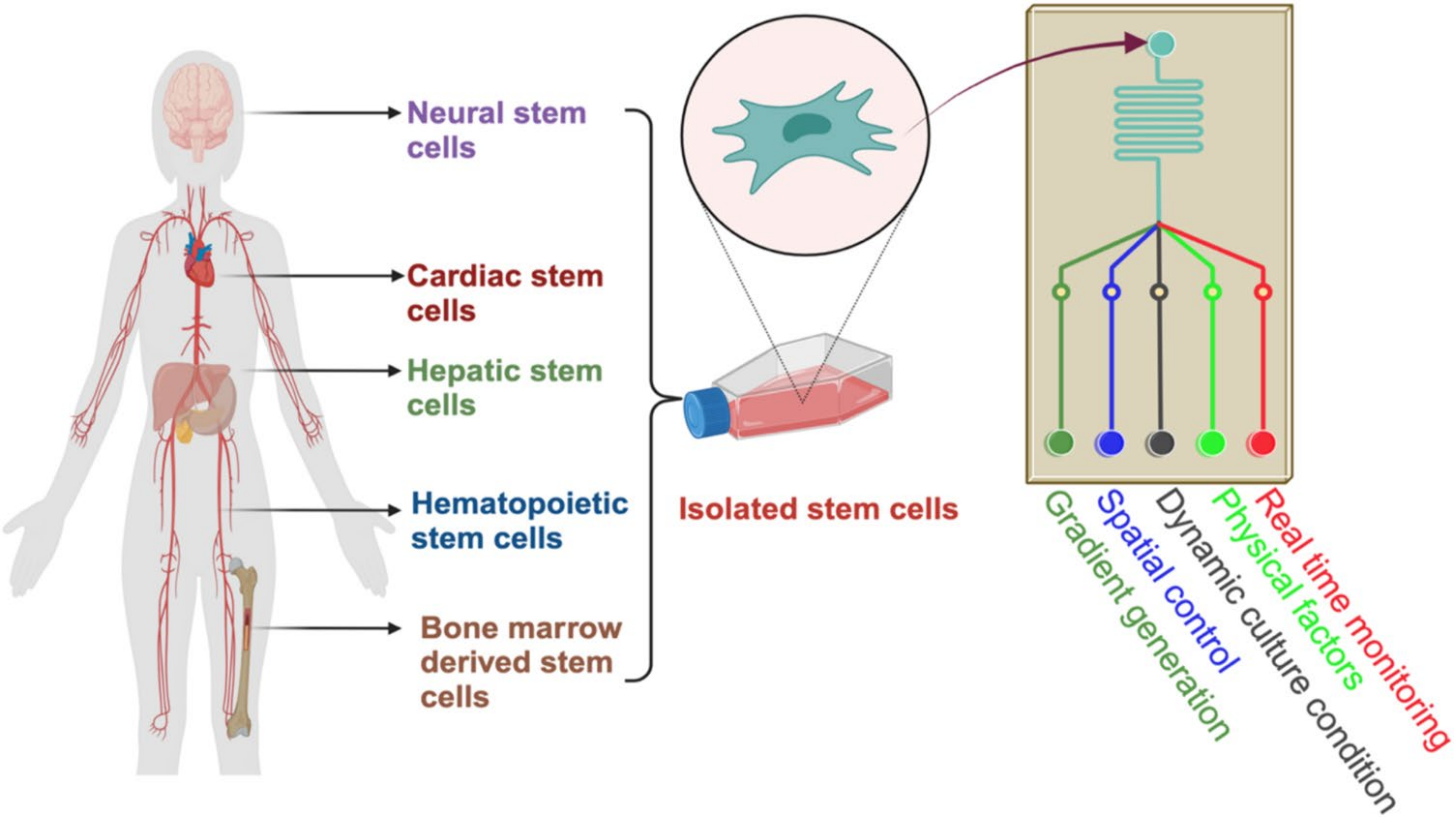
Regulation of stem cell differentiation in microfluidic device

Stem cell differentiation in microfluidic environments is a rapidly growing field that focuses on utilizing microfluidic platforms to control and guide the differentiation of stem cells into specific cell types. Microfluidic systems offer unique advantages for studying and influencing stem cell differentiation due to their ability to provide precise control over various microenvironmental factors (Fig. 4).

### Gradient generation for differentiation

One essential aspect of stem cell differentiation in microfluidic environments is gradient generation for differentiation [82]. Microfluidic devices can create controlled chemical gradients within microchannels, which is particularly useful for directing stem cell differentiation by exposing cells to varying concentrations of signaling molecules or growth factors. A microscale cell stimulator has been developed to administer mechanical, electrical, and biochemical stimulations independently or in combination. Mechanical stimulation induces changes in the actin cytoskeleton of human bone marrow mesenchymal stem cells [97].

### Spatial Control and Patterning

Another critical aspect is spatial control and patterning. Microfluidic platforms enable the spatial control of cell placement and culture through microfabrication techniques that create precise patterns of cells or extracellular matrix proteins. This technique influences differentiation based on spatial cues and promotes cell–cell interactions that play a crucial role in the differentiation process [98]. A recent study has presented a microfluidic approach that utilizes engineered signaling centers to control the self-organization of human pluripotent stem cell colonies. Spatiotemporally controlled morphogen gradients generated from these signaling centers help produce distinct and axially arranged differentiation domains in response to localized sources of bone morphogenetic protein 4 (BMP4). The study also highlights the effects of morphogen concentration and cell density on the BMP response and germ layer patterning [99].

### Dynamic culture conditions

Dynamic culture conditions are also of great importance when studying stem cell differentiation in microfluidic environments. Continuous perfusion of culture media in microfluidic systems ensures a constant supply of nutrients and oxygen, mimicking the dynamic nature of in vivo environments [100]. This dynamic culture condition can influence stem cell differentiation by providing more physiological cues. An article in the literature describes two microfluidic strategies, static and dynamic, to expose human pluripotent stem cells to differentiation-inducing extracellular signals, resulting in a regionalized culture with differentiated cells on one side and undifferentiated cells on the other [101].

### Physical factors

Microfluidic systems can also apply controlled shear stress to cells, mimicking the mechanical forces present in certain tissues. Endothelial cells derived from human pluripotent stem cells (hPSC-ECs) display high sensitivity to low shear stress levels, necessitating prolonged exposure to induce stable phenotypic alterations. This resulted in elevated expression of arterial markers, indicating the transformation of hPSC-ECs into an arterial phenotype [102]. This mechanical stimulation can affect stem cell differentiation outcomes. The mechanical aspect of the stem cell microenvironment is a fundamental component, encompassing the mechanical assistance provided by the extracellular matrix (ECM), the forces applied to cells by their surroundings, and the forces generated through cell interactions with adjacent supporting cells [103]. In vivo, the processes of development, growth, proliferation, and differentiation of stem cells are intricately linked to the mechanical conditions within their surrounding environment.

### Real-time monitoring and analysis

Monitoring real-time stem cell differentiation demands non-invasive, non-destructive, and label-free sensing methodologies. Traditional analysis techniques like immunocytochemistry, polymerase chain reaction, and Western blot are invasive, intricate, and time-consuming. In contrast, electrochemical and optical sensing approaches offer non-invasive qualitative identification of cellular phenotypes and enable quantitative analysis of stem cell differentiation, presenting an innovative alternative to conventional cellular sensing methods.

The existing literature describes a study that proposed the development of an electrochemical sensor. This sensor was based on a droplet microfluidic system that was capable of detecting osteogenic differentiation. The sensor was designed to analyze the differentiation of stem cells, in a non-invasive manner, without the need for a label. This was achieved by detecting the impedance of a single cell. The researchers discovered that the impedance of undifferentiated and differentiated cells differed and that the variation in cell impedance decreased as osteogenic differentiation progressed. This indicates the potential of the electrochemical sensor for detecting and monitoring osteogenic differentiation and highlights its potential for use in the field of stem cell research [104].

A recent investigation proposed a novel microfluidic system-based sensing and cultivation platform designed to monitor the functionality of cardiomyocytes. The platform comprised an aptamer and a gold-based microfluidic system that electrochemically analyzed the cellular function of cardiomyocytes differentiated from induced pluripotent stem cells (iPSCs). To investigate the interaction between cardiac and heart cancer tissues cultured on the platform, the researchers used troponin secreted from each cell to detect single or dual interaction with the platform. This approach provided a unique opportunity to explore the interaction dynamics between different cell types and to monitor cardiomyocyte functionality in vitro [105].

## Challenges and future directions

Stem cell microfluidics has emerged as a promising field for regenerative medicine, but it also presents several challenges that need to be addressed for the full realization of its potential. One of the major challenges is the development of improved stem cell differentiation and integration techniques that can create functional, viable tissues and organs. Moreover, researchers need to enhance their control over the mechanical and chemical properties of microenvironments to better emulate in vivo conditions. The creation of more sophisticated microfluidic devices that accurately mimic the complex microenvironments found in the human body is also necessary.

In the future, stem cell microfluidics is likely to take exciting new directions in the field of regenerative medicine. One such direction is the use of microfluidic devices to create intricate, multi-cellular systems that emulate the structure and function of tissues and organs. This has the potential to revolutionize the field of tissue engineering and lead to the development of new therapies that can repair or replace damaged tissues and organs. Additionally, researchers are exploring the use of stem cell microfluidics in drug discovery, where it has the potential to hasten the identification and development of new treatments for a wide range of diseases. Finally, the use of stem cell microfluidics to study disease progression in vitro is gaining momentum, which could lead to a better understanding of the underlying mechanisms of many diseases and the development of new therapies.

## Conclusion

In the field of regenerative medicine, the convergence of stem cells and microfluidics has emerged as a promising avenue for innovation. Stem cells, with their vast potential for tissue repair and regeneration, and microfluidics, with their precise control over the cellular microenvironment, can be integrated to unlock novel insights into stem cell biology and develop advanced therapeutic strategies. This convergence presents an opportunity to create biomimetic microenvironments and organ-on-a-chip models, which hold immense potential for enhancing healthcare outcomes, from tissue engineering to disease modeling and drug development. As research in this area continues to progress, transformative advancements in medical science can be expected, which will improve the lives of people globally. In conclusion, the integration of stem cells and microfluidics is a promising frontier in regenerative medicine that can revolutionize the way we approach healthcare.

## Data Availability

No datasets were generated or analysed during the current study.
